# The Role of Gut Microbiota in the High-Risk Construct of Severe Mental Disorders: A Mini Review

**DOI:** 10.3389/fpsyt.2020.585769

**Published:** 2021-01-12

**Authors:** Gabriele Sani, Mirko Manchia, Alessio Simonetti, Delfina Janiri, Pasquale Paribello, Federica Pinna, Bernardo Carpiniello

**Affiliations:** ^1^Fondazione Policlinico Universitario “Agostino Gemelli” Istituto di ricovero e cura a carattere scientifico (IRCCS), Rome, Italy; ^2^Section of Psychiatry, Department of Neuroscience, Università Cattolica del Sacro Cuore, Rome, Italy; ^3^Section of Psychiatry, Department of Medical Sciences and Public Health, University of Cagliari, Cagliari, Italy; ^4^Unit of Clinical Psychiatry, University Hospital Agency of Cagliari, Cagliari, Italy; ^5^Department of Pharmacology, Dalhousie University, Halifax, NS, Canada; ^6^Menninger Department of Psychiatry and Behavioral Sciences, Baylor College of Medicine, Houston, TX, United States; ^7^Department of Neurology and Psychiatry, Sapienza University of Rome, Rome, Italy; ^8^Department of Psychiatry, Icahn School of Medicine at Mount Sinai, New York, NY, United States

**Keywords:** microbiome, schizophrenia, depression, genomics, animal models, autism spectrum disorder, Shannon index, alpha diversity

## Abstract

Severe mental disorders (SMD) are highly prevalent psychiatric conditions exerting an enormous toll on society. Therefore, prevention of SMD has received enormous attention in the last two decades. Preventative approaches are based on the knowledge and detailed characterization of the developmental stages of SMD and on risk prediction. One relevant biological component, so far neglected in high risk research, is microbiota. The human microbiota consists in the ensemble of microbes, including viruses, bacteria, and eukaryotes, that inhabit several ecological niches of the organism. Due to its demonstrated role in modulating illness and health, as well in influencing behavior, much interest has focused on the characterization of the microbiota inhabiting the gut. Several studies in animal models have shown the early modifications in the gut microbiota might impact on neurodevelopment and the onset of deficits in social behavior corresponding to distinct neurosignaling alterations. However, despite this evidence, only one study investigated the effect of altered microbiome and risk of developing mental disorders in humans, showing that individuals at risk for SMD had significantly different global microbiome composition than healthy controls. We then offer a developmental perspective and provided mechanistic insights on how changes in the microbiota could influence the risk of SMD. We suggest that the analysis of microbiota should be included in the comprehensive assessment generally performed in populations at high risk for SMD as it can inform predictive models and ultimately preventative strategies.

## Introduction

Severe mental disorders (SMD), including schizophrenia, bipolar disorder and major depressive disorder, are commonly occurring psychiatric conditions exerting an enormous toll on society (1). The 2010 estimate of Gustavsson and co-authors showed that cumulatively direct and indirect costs associated to SMD amount at ~€140 billion per year in Europe (2). Several factors, other than the elevated prevalence in the general population, determine the substantial burden of SMD. First, their longitudinal trajectory start during late adolescence-young adulthood with a life-long duration in the vast majority of cases (3, 4). Second, the clinical course of SMD is often chronic with recurrent episodes of psychopathological disturbances and presence of persistent residual symptoms that significantly affect functioning and quality of life. Indeed, SMD represent a major contributor to the total amount of disability-adjusted life-years attributed to communicable and non-communicable diseases at a global level (5). This appears to be mainly determined by the third determinant of burden, i.e., the presence of suboptimal patterns of response to treatments, either pharmacological or non-pharmacological, leading to only a minority of patients achieving psychopathological and functional remission. Finally, SMD are associated with a considerable excess morbidity and mortality (6–8), which cause a significant reduction in life expectancy (on average 10–20 years) compared to the general population (9, 10). In this context, there has been a constant attempt to improve outcomes of SMD. This strategy has mainly focused on prevention, with the most validated paradigm focusing on primary prevention in individuals presenting subtle symptoms and at clinical high risk for SMD (11). Although the early phases of SMD appear to have distinct developmental trajectories for major affective disorders (4) and schizophrenia (3), particularly in the prodromal phases, there is a general consensus that individuals at risk for SMD are those having a genetic liability due to a high familial loading and/or the presence of antecedents such as basic symptoms, cognitive development, affective lability, anxiety, sleep problems, and psychotic-like experiences (11–13).

In this context, risk prediction of SMD is of paramount importance. Several modeling approaches have been developed using clinical (phenotypic) (14), genomic (15, 16), epigenomic (17), or integrated phenotypic-omics datasets (18). However, although the accuracy of prediction in the proposed models appears adequate for clinical purposes (18), and/or feasible in their implementations (14), there is still need of replication and validation of their predictive power in real life clinical settings. One biological component, partly inherited (19), that has been so far neglected in risk prediction of SMD, is the microbiota. The human microbiota consists in the ensemble of microbes, including viruses, bacteria, and eukaryotes, that inhabit several ecological niches of the organism (20, 21). Due to its demonstrated role in modulating illness and health, much interest has focused on the characterization of the microbiota inhabiting the gut (20). In fact, alterations of the gut microbiota have been linked, among the others, to obesity (22), maturation of the immune system (23), and response to drugs (24). Of particular interest is the modulating role that the microbiota acquires in human behavior (25), raising the interest for the investigation of its modifications in SMD. Indeed, several studies have shown substantial alterations, mainly decreased diversity in species within the microbiota, in schizophrenia (26, 27), in bipolar disorder (28), and major depressive disorder (29, 30). For instance, Zhu and coauthors found that, compared to 81 healthy controls, the gut microbiota of 90 medication-free patients with schizophrenia harbored many facultative anaerobes such as *Lactobacillus fermentum, Enterococcus faecium, Alkaliphilus oremlandii*, and *Cronobacter sakazakii/turicensis*, typically rare in a healthy gut (31). Of note the schizophrenia-associated bacterium *Streptococcus vestibularis*, which contributed to the microbiota metagenomic-based discrimination of patients with schizophrenia from healthy controls, when transplanted to mice gut induced deficits in social behaviors, altering neurotransmitter levels in peripheral tissues of recipient animals (31). In bipolar disorder, Painold and co-authors found that gut microbiota alpha-diversity decreased with increasing illness duration and that Actinobacteria and Coriobacteria were overrepresented in patients compared to healthy controls (HC) (28). Finally, patients with major depressive disorder showed a statistically significant overrepresentation of Bacteroides enterotype 2 compared to controls (32). In addition, a recent systematic review showed that gut dysbiosis and the leaky gut may affect pathways implicated in the neurobiology of major depressive disorder, such immune regulation, oxidative and nitrosative stress, and neuroplasticity (29). However, there is still limited evidence on how microbiota might vary in individuals at risk for SMD compared to healthy controls, as well as to individuals in later stages of SMD. However, there is extensive evidence that the microbiota has a key role in neurodevelopment and can be a modulating factor of the maturity of the central nervous system (CNS) in early developmental stages (33). In this scenario, the aim of this mini review is to present the current evidence on microbiota changes in individuals at high risk for SMD, offering a developmental perspective and providing mechanistic insights on how changes in the gut microbiota make-up could influence the risk of SMD.

## Gut Microbiota in at Risk Mental States: A Developmental Perspective

Recent evidence suggests that the shaping of the microbiome occurs in parallel with the growth of CNS and that they have similar critical developmental windows (34). Consequently, the influence of alterations of gut microbiota on brain maturation trajectories, as well as their relationship with an increased risk for mental disorders later in life have been extensively investigated by preclinical studies (35, 36). In fact, alterations in maternal microbiome have been shown to impact offspring's brain maturation and post-natal development of psychopathology. Buffington et al. (37), observed that the offspring of high-fat diet exposed mice showed autism spectrum disorders/schizophrenia-like symptoms, such as reduced social interactions, poor interest in social novelty, and altered sociability compared to the offspring of normal fed mice (37). These behavioral alterations were coupled with a 9-fold reduction of *Lactobacillus reuteri* and a reduced number of cells producing oxytocin in the paraventricular nuclei of the hypothalamus (37). Other studies investigated the effect of altered maternal gut microbiome on the offspring's behavior through the administration of antibiotics during or immediately before mice pregnancy. A plethora of postnatal aberrant behavior, such as decreased locomotor and explorative activity, low prepulse inhibition, poor social interactions, and anxiety emerged (38, 39). Interestingly, aberrant behavior was completely reversed after fostering the pups by control dams (39). Other factors, such as maternal exposure to stress, can alter the offspring's gut microbiome and affect behavior. Several studies showed that the offspring exposed to perinatal maternal stress showed decreased levels of *Lactobacillus and Bifidobacterium* (40–42). These alterations were associated to increased anxiety and impaired cognitive functions, which started early during development and lasted until adulthood (40–42). Furthermore, gut microbiome composition and behavioral alterations were paired with increased levels of interleukin-1β and decreased brain-derived neurotropic factor (BDNF) in the amygdala (41).

Together with the intrauterine stage, the postnatal period represents a critical moment for both gut microbiota and brain development (34). This developmental stage represents the time when the most dramatic changes in the composition of the intestinal microbiota take place. These are mainly driven by a series of factors, spanning from maternal delivery modalities to genetic diathesis (43–45). Therefore, the interactions between the developing gut microbiota and brain structure and function in this specific developmental phase have undergone extensive investigations. Sudo et al. reported that germ-free (GF) mice, i.e., animals that have never had contact with any microorganism, showed heightened hypothalamic-pituitary-adrenal (HPA) system response to acute restraint stress as compared to mice with a normal gut flora (46). Such phenotype was accompanied by reduced expression of hippocampal and cortical brain-derived neurotrophic factor (BDNF). When GF were administered with a single strain of bacterium*, Bifidobacterium infantis*, stress response normalized (46). However, normalization processes were only possible in GF at early developmental stage, whereas the same procedure in later stages had no effects (46). Another study (47) demonstrated that GF mice showed anxious behavior and increased levels of serotonin in the hippocampus. Even in this case, gut colonization after weaning, which is comparable to adolescence in humans, was uncapable of restoring normal serotonin levels, even though anxiety normalized. Accordingly, in another study (48), post-weaning bacteria colonization was not able to normalize myelin oligodendrocyte glycoprotein levels in GF mice. Cumulatively, these data point toward the existence of specific, and limited, critical periods for the gut microbiota to act on neuronal circuits function and plasticity. The work of Desbonnet et al. (49) further expanded such concept. In their work, post-weaning colonization only partially corrected autism-spectrum-disorder-like behavior in GF mice: self-grooming and social avoidance improved, whereas social cognition did not (49). The authors suggested that the window of opportunity for the microbiota to impact brain circuits might be different for distinct emotional/social behaviors and, eventually, sensory modalities (49). These findings are summarized in Table 1.

**Table 1:** Summary of findings of the pre-clinical studies and/or postulated biological underpinnings of SMD on gut microbiota.

**Animals** **(gender, age)**	**Tested hypothesis (SMD)**	**Preclinical model**	**Tested biological correlates**	**Findings/results**	**References**
GF, SOF BALC/C mice (males, 9–17 weeks old)	Microbiome influence on the HPA stress response (N/A)	Acute restraint stress, ether stress	Plasma ACTH, CRT, IL-1β and IL-6 bioactivity^*^; assessment of fecal microbiota through culture; RT-PCR for CRH, GR, NMDAR gene expression on CTX, HPC, HPT; ELISA for BDNF, NT-3, NGF on HPC and HPT.	Higher ACTH and corticosterone plasma levels in response to restraint stress among GF mice as compared to SPF, but not in response to ether stimulation. Lower BDNF expression among GF in CTX and HPC tissues as compared to SPF. Normalization of the HPA stress response with an early reconstitution of the gut microbiome with Bifidobacterium infantis; an increased stress response was observed with enteropathogenic *Escherichia coli*, but not with a strain devoid of the translocated ITR gene.	(46)
GF, SPF NMRI (males, 8–10 weeks old)	Gut microbiome influence on normal brain development and behavior (N/A)	Open field test, Light-Dark Box test, Elevated Plus Maze test	NA, MHPG, DA, DOPAC, HVA, 5-HT, and 5-HIAA on CTX, HPC, STR tissue assessed through RPHPLC; assessment of the NGFI, BDNF, DR1, DR2, DARPP-32 expression with ISH on AMG, HPC, CTX samples; SNP, PSD-95 assessed through WB on CTX, STR, HPC.	GF showed altered expression of genes involved in second messenger pathway and synaptic potentiation, as well as increased motor activity and lower anxiety behaviors as compared to SPF. Early exposure of GF to gut microbiota resulted in a normalization of GF locomotor activity; adult exposure to gut microbiota failed to normalize GF behaviors. GF presented higher expression of SNP, PSD-95 in the STR as compared to SPF. Higher turnover rates were observed among GF for NA, DA, 5-HT in STR as compared to SPF. GF subjects presented lower BDNF expression in the HPC, AMG, CTX, and lower expression of NGFI-A in the STR, CTX, HPC as compared to SPF.	(35)
GF, CC Swiss Webster (males and females, 6–9 weeks old)	Sex differences in the gut microbiome regulation of the hippocampal serotonergic system (N/A)	Novel-environment stress	Plasma CRT; 5-HIAA, 5-HT, KYNA assessed through HPLC; TNF-α following LPS splenocyte stimulation;	GF presented a lower TNF-α production following LPS stimulation and a higher CRT response to stress as compared to CC regardless of gender; male GF subjects presented lower BDNF, and higher production of 5-HT and 5-HIAA in the HPC as compared to CC, as well as a higher plasma TRP and a decreased KYNA/TRP ratio; GF female had a lower body weight as compared to CC. Gut microbiota recolonization led to a normalization of TRP concentration and of anxiety-like behaviors; no effect was described on the 5-HT and 5-HIAA concentrations in the HPC.	(47)
GF, CC Swiss Webster (males and females, 7–8 weeks old); NIH Swiss strain as stimulus mice in the tests	Gut microbiota influence on social behaviors (Autism)	Sociability and social novelty preference (three chamber test); social transmission of food preference test	N/A	-Social impairment among GF as compared to CC (i.e., more time spent in an empty chamber instead of one shared with another subject); GF did not spend more time analyzing unfamiliar environment over familiar ones, as compared to CC. - A second cohort confirmed social deficits and reduced preference for social novelty among GF; the post-weaning bacterial colonization resulted in the reversal of the observed social aversion but did not affect social cognition impairments. GF spent more time in repetitive self-grooming behaviors and less time in social investigation during the social transmission of food preference test; these behaviors normalized after gut microbiota colonization.	(49)
BALB/C mice VPA-E and CON (VPA-E mice were exposed *in utero* at G11; males and females, 4 weeks old)	The association between altered gut microbiota and autism-like behaviors (Autism)	Social behavior scores (time spent near unfamiliar gender- matched mouse)	Cecal levels of SCFA (i.e., acetic, propionic, butyric, isobutyric and valeric acids); 16S rRNA analysis on cecal samples to investigate the stool bacteria composition	Butyric acid levels were higher among male VPA-E mice as compared to CON. No difference was found for the other SCFA assessed. OTU expression was significantly influenced among VPA-E males; changes observed in the gut microbiota correlated with increased ileal neutrophil infiltration, increased intestinal butyrate level, a reduced level of intestinal serotonin and lower social behaviors score.	(36)
SD rats, PNS-E and CON (PNS rats were exposed to restraint stress during gestational day 14–20; males, 2–4 months old)	The complex interplay between prenatal stress, major physiological systems and gut microbiota composition	Behavioral screening (Open field, elevated plus maze, novel object recognition); acute restraint test	- 1st cohort: colon excision and analyzed for innervation density (confocal fluorescence imaging), and secretomotory function (chambers) - 2nd cohort: 16S rRNA analysis on fecal samples to investigate the stool bacteria composition; tail-bleed plasma corticosterone assessment following acute restraint test, somatic pain sensitivity with the hot plate test and respiratory function (whole body plethysmography) - 3rd cohort: blood pressure, colorectal distension, acute restraint stress	PNS-exposure resulted in a decreased distal colon innervation and an increased secretory response to cathecholaminergic stimulation; PNS-exposed rats presented a lower expression of Lactobacillus and a higher expression of Oscillibacter, Anaerotruncus and Peptococcus. The observed changes in the gut microbiota correlated with respiratory and HPA-axis changes.	(40)
Wistar rats, SST-E and CON (SST-E rats were exposed to an unabsorbable antibiotic starting 1 month before breeding until gestational day 15; male and female, 3–7 weeks old)	The complex interplay between perinatal antibiotic exposure and the offspring mental health	Behavioral screening (open field, social interactions, marble burying, elevated plus maze, prepulse inhibition of the acoustic startle reflex)	Homocysteine and tryptophan levels among untested siblings and dams (preconceptional and post euthanasia levels)	No abnormality was documented in the homocysteine and tryptophan levels between SST-E and CON, ruling out folate deficiency in the SST-E group. SST-E showed decreased social interactions, increased anxiety behaviors (i.e., reduced exploration of the open arm in the elevated plus maze), and altered sensorimotor gating (i.e., reduction in the startle inhibition)	(38)
C57/B16 mice, PNS-E and CON (PNS-E mice were exposed during gestational day 10–16; females, 8–10 weeks old)	The complex interplay between perinatal stress, commensal microbes and anxiety-like behaviors in the female offspring	Behavioral screening (elevated plus maze, novel object recognition test, tail suspension test)	- 1st cohort: euthanized at the 17th gestational day for tissue collection - 2nd cohort: behavioral testing, parturition, microbiome sampling (16S rRNA), tissue collection from offspring (i.e., IL-1β, BDNF in placental, and in both fetal and maternal brain)	Stress exposure influenced the maternal gut microbiota; no significant difference was found in the placental microbiota composition. PNS-E mice presented higher Bacteroides and Firmicutes expression as compared to CON; at the family level, a relative increase of the Bifidobacteriaceae, and RIkenellaceae was described. Prenatal stress exposure resulted in increased anxiety-like behaviors and neophobia. Stress exposure resulted in reduced BDNF placental levels and higher levels of IL-1β in placental and fetal brain tissues; adult PNS-E mice had lower BDNF levels in the amygdala.	(41)
GF and CC Swiss Webster (males and females, 10 weeks old)	Gut microbiota influence on prefrontal cortex myelination	N/A	RNA-sequencing, qRT-PCR within various brain regions to investigate myelin component genes; protein extraction and western blot; transmission electron microscopy on prefrontal cortex samples (gathered from 6 male mice)	Differential expression of 250 genes between GF/colonized-GF and CON (14 out of the 94 upregulated genes in GF were directly involved in myelinization, but none of them were upregulated in the colonized-GF); GF and colonized-GF differed for the expression of 15 genes. qRT-PCR confirmed abnormal expression of five myelin component genes among GF; the increased in mRNA expression was confined to the prefrontal cortex, and the gut colonization resulted in the normalization of the genes expression. Electron microscopy revealed increased myelinization among GF regardless of axonal diameter; normalization of mRNA transcription with colonization did not result in a reduction in the relative myelin protein abundance.	(48)
SPF C57BL/6 mice UAA-E and CON (UUA mice were exposed to an unabsorbable antibiotic *in utero* during gestational day 9–16; males and females, 4 weeks old)	The effects of antibiotic exposure on offspring behaviors	Open field test, social interaction test (three chambers social test), 24 h home cage activity test. Study subjects divided in 3 cohorts:-1st cohort: exposed to UAA *in utero* and fostered by CON-2nd cohort: not exposed to UAA *in utero* and fostered by CON-3rd cohort: not exposed to UAA *in utero* and fostered by CON	16S rRNA analysis on fecal samples to investigate the stool bacteria composition	T-RFLP demonstrated different gut microbiota expression between UAA exposed dams and control dams; different expression of gut microbiota was reported between UAA and CON groups also in the offspring. UAA offspring presented lower body weight, lower activity levels in the dark phase of the 24 h home cage activity test, reduced locomotor activity in the open field test, and reduced rearing behaviors in a novel environment. The 1st and the 2nd cohort presented a similar phenotype at week 4 and differed significantly from the 3rd cohort.	(39)
SPF C57BL/6, GF, ASF (males and females, 6–10 weeks old)	Investigating the role of gut microbiota in microglia maturation process	N/A	16S rRNA analysis on fecal samples to investigate the stool bacteria composition; LMCV challenge through right hemisphere injection; LPS challenge applied intracerebrally and intraperitoneally under anesthesia; RT-PCR on adequately processed FACS-separated microglial cells to analyze gene expression; histology IHC and three-dimensional microglia reconstruction	Different mRNA expression between SPF and GF mice was observed, especially among genes involved in cell activation, in pathogen recognition and host defense regulation. Flow cytometry allowed to recognize a pattern consistent with immature microglia phenotype. GF presented more Iba-1+ microglial cells featuring longer processes, more segments and establishing more physical contacts with adjacent cells as compared to SPF. LPS challenge and LCMV test revealed an abnormal immune response among GF subjects, heralded by differential expression of genes involved in the immune response and prominent morphological anomalies. Antibiotic exposure for 4 weeks, induced similar phenotypic changes in microglial cells among SPF, but with no changes in cell numbers; ASF tri-colonized (*Bacteroides distasonis, Lactobacillus salivarius*, Clostridium cluster) presented both increased microglial cell numbers and morphological changes, despite having near-normal biomass, reversible by allowing a more diverse bacterial colonization with SPF co-housing. Intriguingly, a 4-week course of SCFA supplements resulted in the normalization of the microglial phenotype among GF.	(50)

* *IL-6 bioactivity assessed through IL-6-dependent B cell hybridoma*.

*5-HIAA, 5-Hydroxyindoleacetic acid; 5-HT, 5-Hydroxytryptamine; ACTH, Adrenocorticotropic hormone; AMG, Amygdala; ASF, Altered Schaedler flora; BDNF, Brain Derived Neurotrophic Factor; CC, Conventionally Colonized; CRH, Corticotropic Releasing Hormone; CON, Controls; CRT, Corticosterone; CTX, Cortex; GF, Germ Free; DA, Dopamine; DARPP-32, Dopamine- and cAMP-regulated phosphoprotein Mr 32 kDa; DOPAC, Dihydroxyphenylacetic acid; DR1, Dopamine Receptor 1; DR2, Dopamine Receptor 2; G11, Gestational day 11th; GF, Germ Free; GR, Glucocorticoid Receptor; HPA, Hypothalamic Axis; HPC, Hippocampus; HPLC, High-Performance Liquid Chromatography; HPT, Hypothalamus; HVA, Homovanillic acid; IHC, Immunohistochemistry; ISHT, In Situ Hybridation; ITR, Intimin Receptor; KYNA, Kynurenic acid; LCMV, Lymphocytic Choriomeningitis Virus; LPS, LipoPolySaccharide; MHPG, 3-Methoxy-4-HydroxyPhenylGlycol; N/A, Not Available; NA, noradrenaline; NGFI-A, Nerve Growth Factor-Inducible clone A; NMDAR, N-Methyl- D - Aspartic Acid Receptor subunits (NR-1 and NR-2A); NGF, Nerve Growth Factor; NT-3, Neurotrophin-3; OTU, Operational taxonomic unit; PNS-E, Prenatal stress in utero exposure; PSD – 95, Postsynaptic density protein 95; qRT-PCR, quantitative Real Time Polymerase Chain Reaction; RPHPLC, Reversed-Phase High-Performance Liquid Chromatography; SCFA, Short chain fatty acids; SD, Sprague-Dawley; SMD, Severe Mental Disorder; SNP, Synaptophysin; SPF, Specific Pathogen Free; SST-E, Succinyl Sulfa Thiazole exposed in utero; STR, Striatum; TNF-α, Tumor Necrosis Factor-α; T-RFLP, Terminal Restriction Fragment Length Polymorphism; UAA, Unabsorbable Antibiotic; UAA-E, Unabsorbable Antibiotic exposure in utero; VPA-E, Valproic Acid in utero Exposure; WB, Western Blotting*.

## Gut Microbiota in at Risk Mental States: Clinical Data

Despite the relatively large amount of studies investigating the relationship between gut microbiota composition and neurodevelopmental alterations in mice, only one study investigated the effect of altered microbiome and risk of developing mental disorders in humans (51). Specifically, He et al. (51) investigated alpha-diversity (i.e., the bacterial diversity within a single sample) and beta-diversity (differences in species composition among samples) metrics of gut microbiome in high-risk (HR), ultra-high-risk (UHR) subjects for developing schizophrenia and HC (51). Beta-diversity analysis revealed that UHR and HR had significantly different global microbiome composition than HC. Furthermore, UHR showed greater levels of *Clostridiales, Lactobacillales, Bacteroidales*, higher levels of Acetyl coenzyme A synthesis and greater anterior cingulate choline levels than the both HR and HC. The authors pointed out that the alterations in microbiome overlapped with those identified in schizophrenia and autism-spectrum disorder (52, 53). Additionally, higher levels of choline were interpreted as resultant of altered membrane metabolism due to microglial activation, which is one of the possible mechanisms mediating the effects of an altered gut microbiome on neural development (51). Putative mechanisms of the interplay between microbiota and genetic predisposition in modulating the liability toward the development of a SMD is discussed below. We have summarized clinical evidence in Table 2.

**Table 2:** Summary of findings of the clinical studies on gut microbiota in SMD.

**SMD (diagnostic criteria)**	**Sample size and composition (Age range; gender composition)**	**Methods**	**Findings/results**	**References**
AD, PDD (DSM – IV)	AD *n* = 10, PDD-NOS *n* = 10, HC *n* = 10 (4–10 y.o.; 14 M, 16 F)	Cross-sectional study; ADI-R, ADOS, FDO; 16S rDNA and 16S rRNA analysis on fecal samples to investigate the stool bacteria composition, its metabolic activity and an assessment of the organic volatile compounds and free fatty acids composition.	PDD-NOS and HC presented higher *Faecalibacterium* and *Ruminococcus* expression; PDD-NOS and HC presented higher expression of Caloramator, Sarcina, and Clostridium; PDD-NOS and AD presented different composition of Lachnospiraceae as compared with the HC. Different levels of organic volatile compounds and free fatty acid between the three groups.	(52)
HR, UHR (DSM – IV)	HR *n* = 81; UHR *n* = 19; HC *n* = 69 (13–30 y.o.; HR 41 M, 40 F; UHR 15 M, 4 F; HC 37 M, 32 F)	Cross-sectional study; 1H-MRS; APSS, BIPS, GAF-M, GRDS, SIPS, SOPS; HR and UHR were screened for the absence of DSM – IV coded diagnoses; 16S rRNA analysis on fecal samples to investigate the stool bacteria composition.	Increased expression of Clostridiales, *Lactobacillales* and Bacteroidales in UHR compared to the other two groups; increased choline levels on 1H-MRS among UHR subjects compared to the other groups.	(51)
SCZ (ICD-10)	SCZ *n* = 64, HC *n* = 53 (18–65 y.o.; 36 M, 28 F in SCZ; 35 M, 18 F in HC)	Cross-sectional study; 16S rDNA and 16S rRNA analysis on fecal samples to investigate the stool bacteria composition; PICRUSt analysis to probe metabolic pathways; PANSS.	SCZ patients presented higher expression of the Proteobacteria phylum, and at the genus level, a relatively higher expression of Succinivibrio, Megasphaera, Collinsella, Clostridium, Klebsiella, Methanobrevibacter, and a lower of Blautia, Coprococcus, Roseburia as compared to HC; differences in numerous metabolic pathways between HC and SCZ (e.g., fatty acid, vitamin B6).	(53)
GP reported depression (NA)	Subset of the FGFP cohort *n* = 1,054 - GPRD *n* = 80, HC *n* = 70, validated in LLD data sets *n* = 1,070 and in TR-MDD^*^n = 7 group. (FGFP m.a. 50.9, 478 M, 576 F; LLD m.a. 57.9 y.o., 447 M, 616 F; TR-MDD balanced to the FGFP group)	Cross-sectional study; BMI; BSS; GP reported depression, HAM-D; RAND-36; 16S rRNA analysis on fecal samples to investigate the stool bacteria composition.	Butyrate-producing *Faecalibacterium* and *Coprococcus* bacteria were associated with higher QOL. Dialister, *Coprococcus* spp. depletion was observed in depression; microbial synthesis of 3,4-dihydroxyphenylacetic acid appeared positively correlated with mental QOL.	(32)
Bipolar Disorder (DSM-IV)	BD *n* = 32; HC *n* = 10 (BD 20–65 y.o., 18 M, 14 F; HC NA y.o., 4 M, 6 F)	Cross-sectional study; BDI-II; HAM-D; inflammatory markers, serum lipids, KYNA, oxidative stress and anthropometric measures; 16S rRNA analysis on fecal samples to investigate the stool bacteria composition.	BD illness duration was negatively correlated with microbial alpha diversity. Actinobacteria and Coriobacteria were more abundant in BD as compared with HC; Ruminococcaceae and *Faecalibacterium* were more abundant in HC as compared with BD. Certain bacterial clades were more commonly observed with the metabolic and inflammatory patterns observed among BD individuals.	(28)
Schizophrenia (DSM-IV)	90 SCZ, 81 HC, validated in a verification sample 45 SCZ^1^ and 45 HC^1^ (SCZ 14–53 y.o., 46 M, 44 F; HC 18–64 y.o.,41 M, 40 F)	Cross-sectional; MWAS to characterize gut microbiota; MCCB; PANSS; KYNA and tryptophan blood levels; 16S rRNA analysis to probe mice stool microbiota composition.	Different tryptophan and KYNA blood levels between SCZ and HC; SCZ gut microbiota featured higher expression of facultative anaerobes and oral cavity bacteria as compared with HC. Transplantation of Streptococcus vestibularis in mice resulted in altered neurotransmitter production and social behaviors.	(31)

* *TR-MDD: TR-MDD was defined as a diagnosis of either Major Depressive Disorder or Bipolar Type II according to the DSM-IV criteria. 1H-MRS, Proton Magnetic Resonance Spectroscopy; AD, Autism Disorder; ADI-R, Autism Diagnostic Interview-Revised; ADOS, Autistic Diagnostic Observation Schedule; APSS, Attenuated Positive Symptom Syndrome; BIPS, Brief Intermittent Psychotic Syndrome; BDI-II, Beck Depression Inventory; BMI, Body Mass Index; BSS, Bristol stool scale; DSM – IV, Diagnostic and Statistical Manual of Mental Disorders IV edition; F, Female; FDO, Free Direct Observation; FGFP, Flemish Gut Flora Project; GAF-M, General Assessment of Functioning – Modified version; GP, General Practitioner; GPRD, General Practitioner Reported Depression; GRDS, Genetic Risk and Deterioration Syndrome; HAM-D, Hamilton Depression Rating Scale; HC, Healthy Control; KYNA, Kynurenic Acid; LLD, Dutch LifeLines DEEP; M, Male; m.a., mean age; MCCB, MATRICS Consensus Cognitive Battery; MWAS, Metagenome-Wide Association Study; NA, Not Available; n, total size; PANSS, Positive and Negative Syndrome Scale; PDD-NOS, Pervasive Developmental Disorder – Not Otherwise Specified; PICRUSt, Phylogenetic Investigation of Communities by Reconstruction of Unobserved States; QOL, Quality Of Life; RAND-36, RAND-36 health-related quality of life survey; SCZ, Schizophrenia; SMD, Severe Mental Disorder; SIPS, Structured Interview for Prodromal Syndromes; SOPS, Scale of Prodromal Symptoms and fulfilled one of the three subsets; spp, species; TR-MDD, Treatment Resistant Major Depressive Disorder; y.o., years old*.

## Mechanistic Hypotheses on the Influence of Gut Microbiota on at Risk Status for Severe Mental Disorders

There is compelling evidence that the products of gut microbiota might influence behavior in mammals through the action of their byproducts on the CNS (25). For instance, metabolic waste products of the gut microbiota such as the short-chain fatty acids (SCFAs) can influence neuromodulation via inhibition of the histone deacetylases (25, 54). In addition, another byproduct such as butyrate helps maintaining the integrity of the blood-brain barrier (25, 55), while acetate appears to exert anorectic effects via preferential accumulation in the hypothalamus (56). Other sets of findings have pointed to the link between gut dysbiosis and increased gut permeability and alterations of mitochondrial function, with significant repercussions at the CNS level (57). This amount of evidence, supported by the clinical and preclinical findings on the impact of gut microbiota on neurodevelopment, has fostered several mechanistic hypotheses (58, 59). While an extensive discussion of these mechanisms is out of the scope of the present mini review, we present a synthesis that we reckon as relevant for the high-risk construct of SMD. An altered neurodevelopment due to maternal gut flora modifications might be the resultant of poor regulation of maternal/fetus inflammatory state mediated by the maternal gut microbiome (58). Adequate gut microbial colonization in pregnant mice was associated to expression of regulatory T-cells (Tregs). Tregs normalize systemic levels of proinflammatory cytokines, such as IL-17 and interferon-γ (60), thus maintaining correct inflammatory/non-inflammatory balance. The lack of gut microbiota in GF pregnant mice resulted in a decrease of Tregs, with a general imbalance toward maternal and fetal inflammatory state (60). High levels of proinflammatory cytokines have been shown to induce fetal abnormal cortical development and surge of post-natal autism-like behavior (61). Alteration of maternal gut microbiome might also increase levels of fermentation products (CFAs), namely acetate, propionate and butyrate (62). Indeed, CFAs are capable to massively activate microglia, the immune cells of the CNS playing an important role in CNS homeostasis (50). Microglia activity might initiate/exacerbate the inflammatory cascade leading to the massive release of cytokines as well as to associated alterations in the endothelial permeability, including the blood-brain barrier. Such cascade has been shown to predispose to the development of neurodegenerative disorders, including schizophrenia and Parkinson's disease (59, 63).

Another putative mechanism might involve alterations in neurogenesis and specifically the BDNF which is involved in neural growth and cell survival. As previously shown, the gut microbiota is involved in the expression of BDNF (64). Prenatal/postnatal alterations of the gut microbiota can alter BDNF expression, and these changes could alter maturation trajectories of neural circuitry, leading to the development of SMD (65–68). Furthermore, gut microbiota can modify oligodendrocyte products and affect myelination, particularly in the prefrontal cortex, a brain region involved in attention, memory, emotional learning and critically connected to SMD such as ASD (69), schizophrenia (70), major depressive disorder (71), bipolar disorder (72), and substance abuse (73). Specifically, altered myelination has been related to changes in synaptic formation and function, which could lead to the surge of specific cognitive deficits typically seen in schizophrenia, namely deficits in attention, working memory, and executive function (74).

**Figure 1:**
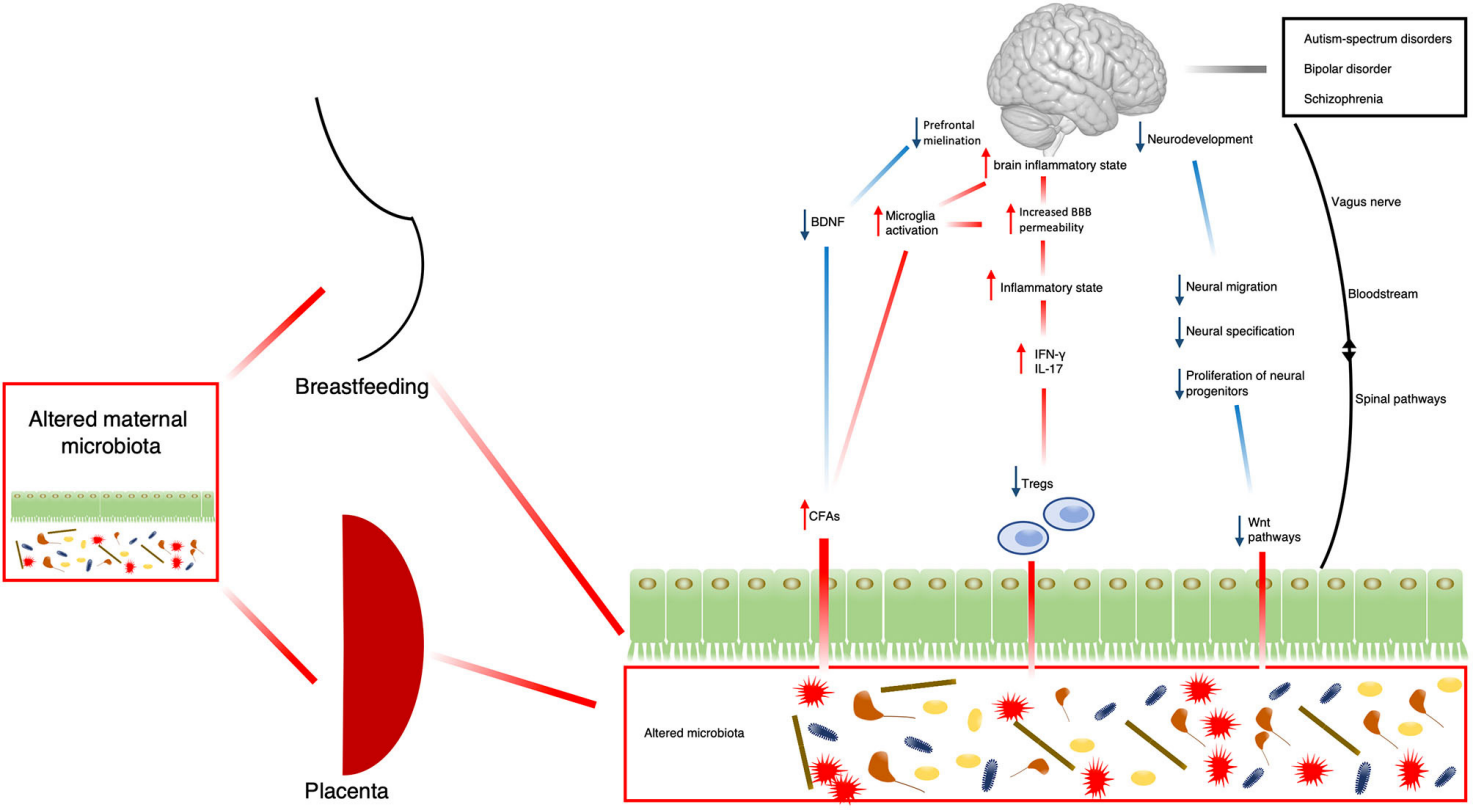
Schematic representation of possible mechanisms by which the microbiota might contribute to the development of SMD. BDNF, brain-derived neurotrophic factor; CFAs, fermentation products; IFN-γ, interferon-γ; IL-17, Interleukin-17; Tregs, regulatory T-cells; Wnt, homologous wingless and Int-1.

Another interesting, but still under-investigated, mechanism is represented by the effect of the gut microbiome on the Wnt pathways. These are signal transduction pathways mainly involved in human development, cell migration and proliferation and tissue regeneration (75). Wnt pathways are also involved in neural morphogenesis, axon guidance, neurite outgrowth, and synaptic plasticity (76, 77). Alterations in Wnt pathways have been recently related to higher risk to develop SMD, such as schizophrenia or bipolar disorder (78, 79). Of note, GF mice showed poor Wnt pathway activity in intestinal stem cells (80), supporting the speculation of a possible link between alteration of gut microglia, altered neurodevelopment and consequent increased risk for SMD. However, proper investigation of the relationship between gut microbiome alterations and altered Wnt pathways is still underdeveloped and needs further research. All these mechanisms are illustrated in Figure 1.

## Conclusions

The findings of our review prompt a series of considerations. First, despite the consensus that microbiota plays a fundamental role in neurodevelopment and substantial changes are detectable in individuals affected by SMD, there is a dearth of studies investigating its modifications during the developmental trajectories of these disorders, particularly in high-risk populations. This could be feasible particularly in consideration that appropriate clinical chemistry and molecular immunology assays to assess for the presence of biological markers of “leaky gut” might be easily implementable in clinical settings (81). Second, only a longitudinal perspective could shed light on the direction of these changes, i.e., whether microbiota modifications precede the onset of psychopathology (of whatever severity) or vice versa. This perspective could be applied, but should not be limited, to the early stages of SMD. Indeed, prospective analysis of microbiota changes are starting to shed light on the longitudinal variation of mood in the course of bipolar disorder (82). Third, this approach can help decrease the confounding associated with the use of drug treatments (if the analyses are performed in pre-diagnostic stages), and at the same time inform on changes that might favor, or be predictive of, response to treatment. In conclusion, we suggest that the analysis of microbiota should be included in the comprehensive assessment generally performed in populations at high risk for SMD as it can inform predictive models and ultimately preventative strategies.

## Author Contributions

MM and GS drafted the first version of the article. AS, DJ, and PP helped with the search of the literature and contributed to the draft of the manuscript. FP and BC oversaw the project and revised the text critically for important intellectual content. All authors gave final approval of the version to be published and agree to be accountable for all aspects of the work.

## Conflict of Interest

The authors declare that the research was conducted in the absence of any commercial or financial relationships that could be construed as a potential conflict of interest.
